# Use of Respiratory Protection Devices by Medical Students during the COVID-19 Pandemic †

**DOI:** 10.3390/ijerph18115834

**Published:** 2021-05-28

**Authors:** Ekaterina A. Shashina, Valentina V. Makarova, Denis V. Shcherbakov, Tatiana S. Isiutina-Fedotkova, Nadezhda N. Zabroda, Nina A. Ermakova, Anton Yu. Skopin, Oleg V. Mitrokhin

**Affiliations:** Institute of Public Health, Department of General Hygiene, I.M. Sechenov First Moscow State Medical University (Sechenov University), 119991 Moscow, Russia; makarova_v_v@staff.sechenov.ru (V.V.M.); shcherbakov_d_v@staff.sechenov.ru (D.V.S.); isyutina-fedotkova_t_s@staff.sechenov.ru (T.S.I.-F.); zabroda_n_n@staff.sechenov.ru (N.N.Z.); ermakova_n_a@staff.sechenov.ru (N.A.E.); skopin_a_yu@staff.sechenov.ru (A.Y.S.); mitrokhin_o_v@staff.sechenov.ru (O.V.M.)

**Keywords:** COVID-19 pandemic, face mask, medical students, coronavirus, respiratory protection devices

## Abstract

The use of face masks has assumed a leading spot among nonspecific prevention measures during the coronavirus pandemic. The effectiveness of this protective measure depends on the specifics of individual use. The purpose of our study was to analyze the use of respiratory protective equipment (RPE) by medical students during the COVID-19 pandemic. The evaluation of face mask use was based on the results of a survey of medical students at Sechenov University. There were 988 participants in the study: 97.5% used RPE during the pandemic, 89.1% used disposable medical and hygienic face masks, 27.4% used reusable cloth face masks, and 13.2% used respirators. The majority of respondents (75.2%) were found to wear face masks correctly. However, 17.0% of the respondents were found to cover only their mouths with a face mask, while 7.8% reported often shifting their face mask under the chin due to perceived discomfort. Only 25.1% of respondents changed their disposable face mask after 2–3 h of wearing, while 13.0% decontaminated and used it several times. Most cloth face mask users (93.7%) decontaminated their marks, but only 55.7% of respondents did so daily. Face masks were most often worn in medical organizations (91.5%), and 1.4% of respondents did not use respiratory protection anywhere. In conclusion, we consider it necessary to introduce a special module on nonspecific prevention in the discipline of hygiene.

## 1. Introduction

The onset of the coronavirus pandemic, in the absence of specific drugs for treatment and prevention, brought to the forefront nonspecific measures to combat this infection, aimed at interrupting the pathways of its transmission. These include temporary general lockdowns on economic and social activities, social distancing, contact tracing and limiting, quarantine, isolation, and hand hygiene, as well as wearing personal respiratory and hand protection equipment [1,2,3,4,5]. Transmission of the pathogen from the source occurs mainly through airborne droplets [1,6]. Therefore, among nonspecific preventive measures, the use of face masks has assumed the leading spot as the most easily implemented measure of individual protection (in comparison with social distancing) [7,8,9]. This measure is recognized by the World Health Organization as effective [10].

In Russia, wearing face masks is a recommendatory or mandatory measure, depending on the sanitary and epidemic situation. The use of face masks by the population is controlled by senior officials of the constituent entities of the Russian Federation (Resolution of the Chief State Sanitary Doctor of the Russian Federation dated 16 October 2020 No. 31 “On additional measures to reduce the risks of the spread of COVID-19 during the seasonal rise in the incidence of acute respiratory viral infections and influenza”). It is recommended to wear face masks in public places, on public transport, in elevators, all service sector facilities, and in healthcare and educational institutions.

Today, at the time of the start of mass immunization of the population, wearing face masks is still relevant as a measure to reduce the risk of contracting the coronavirus. This is due to the rapidly spreading new strains of the virus, which are more infectious than the original one. At the same time, even the creation of specific COVID-19 prophylaxis may not be sufficient to completely stop the spread of the epidemic process. Due to a number of objective and subjective reasons, it is quite difficult to vaccinate a large part of the population in a short time: it is necessary to keep in mind contraindications, chronic diseases, and the negative attitude of some citizens to vaccination. Currently, as the vast majority of the population is not vaccinated, strict adherence to other principles of reducing transmission, that is, face masks and social distancing, remains relevant to reduce the spread of the virus in society [11].

At the beginning of the pandemic, there were difficulties associated with the availability of respiratory protection, because it was our first experience with mass use of face masks. At present, this problem has been solved—a large variety of respiratory protective equipment (RPE) is available to the population of the Russian Federation. Earlier, we reviewed the RPE available in Russia [12] and found that there are four groups of respiratory protection devices: respirators, medical, nonmedical face masks, and face shields. The variety of products included in each group is mainly determined by the materials from which they are made and the number of layers included. Respirators are the most effective when it comes to protecting against viral aerosol, while face masks are effective against airborne transmission of coronavirus infection (a. Russian state standard GOST R 58396-2019. Medical face masks. Requirements and test methods; b. Russian state standard GOST 12.4.294-2015. Occupational safety standards system. Respiratory protective devices. Filtering half face masks to protect against particles. General specifications; c. CEN, E., 2001. 149: 2001 norm: Respiratory protective devices-Filtering half face masks to protect against particles Requirements, testing, marking. European Committee for Standardizationl d. Centers for Disease Control and Prevention. Use face masks to Slow the Spread of COVID-19; Centers for Disease Control and Prevention, 2020. Available online: https://www.cdc.gov/coronavirus/2019-ncov/prevent-getting-sick/diy-cloth-face-coverings.html (accessed on 27 May 2021); e. National Institute for Occupational Safety and Health (NIOSH). NIOSH Guide to the Selection and Use of articulate Respirators. Department of Health and Human Services (DHHS) NIOSH publication number 96-101, 1996) [13].

The effectiveness of this measure of protection against infection also depends on the specifics of individual use of RPE: the way of wearing it (the face mask covering mouth and nose, fitting tightly to the face, or covering only the mouth), the wearing time, and the type and frequency of decontamination of reusable products. Collective protection is determined by the proportion of the population using face masks in daily life. According to Pakistani researchers, the motivations for wearing face masks are “attitude, social norms, risk perceptions of the pandemic, and perceived benefits of face masks are the major influencing factors positively affect public willingness to wear face masks, whereas the cost of face masks and unavailability of face masks tend to have opposite effects” [14]. Compliance with preventive measures by the population, and the wearing of masks in particular, is also influenced by factors such as the region of residence (rural or urban area), housing conditions, sector of employment and education level, age, and income level [9,15,16,17]. American scientists have identified a high risk of COVID-19 infection for all segments of the population. More affluent people are at greater risk because they actively travel and visit places of mass entertainment and sport, but less well-off people are forced to work in person more frequently than remotely and have fewer opportunities to constantly replenish their supply of high-quality personal protective equipment and seek qualified medical care [18]. In some countries, the low socioeconomic status of the population and the lack of preventive tools and official information channels remain factors that reduce the effectiveness of this protective measure [19].

People often refuse to wear face masks because they are embarrassed by their fear of being infected or do not believe in the effectiveness of face masks, and because of ambiguous recommendations by different health authorities [20]. Refusal to wear face masks can often be associated with the inconvenience and problems that arise when wearing them, including skin reactions, headaches, and difficulty breathing [21,22,23]. The almost complete absence of a culture of wearing face masks as a hygienic practice, which is characteristic of the inhabitants of most European countries—in contrast to, for example, the inhabitants of Asia—also contributes to this hesitancy [15,20,21,24]. In addition, the reasons for refusing face masks can be a careless attitude toward one’s own health, as well as a lack of public awareness regarding the effectiveness of using face masks. According to some authors, men are less likely to wear face masks than women [25,26], with women having been found to feel responsible both for themselves and for their children and to make more efforts to preserve the health of the family through measures such as self-isolation, wearing personal protective equipment, healthy nutrition, intrafamily communication, and psychological support [27]. People in some regions (e.g., Thailand, China, Japan) have opted for temporary alternatives or the reuse of disposable surgical face masks. Notably, improper use of face masks, such as not changing disposable face masks, can compromise their protective effect and even increase the risk of infection [20,21].

The COVID-19 pandemic has greatly affected people’s lives, changing their habits and daily lifestyle (communication with relatives and friends, social activity, visiting entertainment events and shopping centers). The state of stress and depression, which are the result of the constant fear for one’s own health and the health of their loved ones, has become more important. In addition, many people have lost their jobs and, consequently, their income, other have been forced to work remotely and, as a result, to work longer hours while mastering new technologies and online platforms, while some sectors of the economy and education have been undergoing a crisis (manufacturing industry, public catering, tourism) [27,28,29,30]. At the same time, though, the quality of atmospheric air has been noted to improve all over the world due to the shutdown of the transport industry, as well as other industries [30].

The current situation requires the mobilization of all members of society to form a conscious attitude toward both the restrictive measures introduced during the pandemic and possible future situations in which wearing RPE in public places is necessary, above all from the position of care for others. Mass media, including social networks and the official electronic resources of government organizations, have an important role to play here, becoming increasingly relevant in situations of isolation [31]. It is important to prevent the spread of rumors, myths, and misinformation regarding the current pandemic, especially among people with low socioeconomic status, because such dissemination can have serious consequences for public health [19]. Some authors recommend paying special attention to information campaigns on COVID-19 aimed at young people—in particular, university students [17].

The leading role in shaping public opinion on the risks associated with the pandemic and the necessary protective measures is assumed by health workers [32], including medical students, as one of the main segments of the population who can lead by example in the formation of an active civil attitude of the population for the prevention of infection. According to Italian researchers, healthcare workers have a sufficient level of information regarding the risks associated with the spread of COVID-19, and they actively use measures of nonspecific protection [33]. Earlier, we conducted a pilot study of medical students’ knowledge and attitudes to the face mask regime [34], which showed that most respondents were civically responsible, understood the importance of using face masks to protect others, and could actively promote their use to the public. The present study is devoted to conducting a further in-depth investigation of the problem of wearing face masks among medical students. They, like all other health workers, are at high risk of infection and can also contribute to the spread of the disease. Therefore, the practice of wearing RPE is most relevant for medical students. The purpose of our study was to analyze the use of RPE by medical students during the COVID-19 pandemic.

## 2. Materials and Methods

The use of face masks was assessed based on the results of the questionnaire. The questionnaire was developed by the staff at the Department of General Hygiene of Sechenov University and distributed among the Russian 3rd-year students at the Institute of Clinical Medicine of Sechenov University in March 2021. The questionnaire was approved by the University Local Ethics Committee. All respondents confirmed their voluntary participation in the survey and consent for the processing of answers.

The questionnaire included the following blocks of questions, which were assigned to two groups of variables during analysis. The first group consisted of independent variables such as gender, year of birth, permanent place of residence within the last 6 months, and status of a medical student. The second group consisted of dependent (categorical) variables such as questions about wearing face masks in various public places, both by the respondent and by others; awareness of the protective properties of RPE; compliance with the rules for their use; and the motivation for their choice during the pandemic.

Students studying in the specialty of General Medicine were chosen for this study because they are more likely than students of other specialties to be exposed to the risks of both becoming infected themselves and infecting others. The 3rd year represents the middle of medical university studies. Thus, students at this point in their academic career have already studied general disciplines and are starting to master special ones, including hygiene. After their 3rd year, students are expected to begin their clinical practice, so knowledge of nonspecific prophylaxis of infectious diseases in a pandemic is especially relevant for them.

The categories of responses were discrete, and the metrizability of the variable space was measured in an ordinal (rank) scale. This characteristic does not limit the use of factor analysis in this study. The reason for the use of factor analysis was the need to determine the relations between variables (to classify variables), as well as to reduce the number of variables necessary to describe the data of this study and conduct further research in this area.

Managing self-selection bias in research was achieved using a continuous questionnaire survey among all 3rd-year students at the Institute of Clinical Medicine of Sechenov University in March 2021 (*n* = 1035). The link to the questionnaire was offered to students by teachers in the classroom and also posted on the united educational portal of Sechenov University on a resource available only to 3rd-year students studying in the specialty “General Medicine”. We received 1014 completed questionnaires, which represents a completion rate of 97.9%. The exclusion criteria were as follows: unfinished completion of the questionnaire by respondents; committing formal errors when completing. In accordance with these criteria, 26 questionnaires were excluded from further analysis. Thus, 988 questionnaires were analyzed by us, which comprised 95.5% of all 3rd-year students studying in the specialty of General Medicine, prompting suggestions that the conditions of the sample generalization principle had been met.

Statistical processing of the research results was carried out using the statistical software package STATISTICA Base (TIBCO, Palo Alto, CA, USA). Frequencies and percentages were computed for categorical variables, and the means and standard deviations were calculated for numerical variables. For frequencies, a 95% confidence interval was calculated using the Wilson method. The significance of the differences in the presented features was determined using the Chi-square test.

The critical value of the significance level (*p*) was taken as *p* ≤ 0.01%.

## 3. Results

The questionnaires of the research participants were analyzed: there were 244 men and 744 women. 

The demographic characteristics of participants are presented in the Table 1.

We confirmed the hypothesis of the absence of statistically significant gender differences in the vast majority of responses (*p* ≥ 0.05). This served as the basis for further analysis of the responses in the combined sample.

Almost all respondents (97.5%) used respiratory protection during the pandemic. The motivation for wearing RPE for 75.8% (73.0–78.5) of the respondents was the introduction of a face mask regime in the region of residence; for 55.5% (52.3–58.7), it was the need to come into close contact with other people; for 51.3% (48.1–54.6), it was the current epidemiological situation; for 43.4% (40.2–46.6), it was requests from relatives and colleagues; for 15.0% (12.9–17.5), it was being in contact with people who do not feel well or exhibit obvious signs of the disease; for 2.8% (1.9–4.1), it was the presence of symptoms of the disease (fever, weakness, respiratory symptoms).

The respondents used different types of RPE: disposable medical and hygienic face masks, reusable fabric face masks/cloth face masks, and disposable respirators. Almost three quarters of the respondents (73.7%) used only one means of protection. In total, 23.2% alternated between 2 different means, whereas 3.1% used all three types of RPE.

Disposable medical and hygiene face masks were the most popular type, preferred by 89.1% (86.9–91.0) of the respondents. They were followed by reusable cloth face masks (27.4% (24.6–30.3)) and disposable respirators (13.2% (11.1–15.5)).

Users of disposable medical and hygiene face masks were more likely to wear 3- and 2-layer products (37.5% and 34.4%, respectively); 1-layer face masks were used by 9.2% of the respondents (18.9% of the respondents did not know the layering of the product used) (χ^2^ = 172.018, *p* < 0.001).

Among reusable products, face masks made of cotton and synthetic fabrics were used almost in equal proportions (45.0% and 42.5%, respectively); 12.5% of the respondents were not aware of the composition of the product used.

For 73.0% (68.9–74.7) of the students, the decisive factor when choosing protective equipment was wearing comfort (no fogging of glasses, no difficulty breathing, etc.). The protective properties of face masks turned out to be less significant (56.6% (53.4–59.8)), along with the possibility of skin reactions to wearing (54.3% (51.0–57.4)), a low price or the possibility of receiving the RPE free of charge (36.6% (33.6–39.8), and the appearance of the protective equipment (17.9% (15.6–20.6)). 

Only 27.3% of survey participants paid attention to the information provided by the manufacturer while purchasing RPE. The most important information for them was the wearing time (62.0% (55.8–67.8)), the composition of the product (58.2% (51.9–64.2)), and the registration of the product in the register of the Federal Service for Surveillance in Healthcare of Russia (49.4% (43.3–55.6)).

The majority of respondents (75.2%) were found to wear a face mask correctly, with the mask covering their mouth, nose, and chin and fitting tightly to their face. However, 17.0% of the respondents reported only covering their mouths with a face mask, while 7.8% often shifted their face mask under the chin due to perceived discomfort (χ^2^ = 1187.9, *p* < 0.01).

The rules regarding the recommended duration of wearing a disposable face mask were followed less carefully. Only 25.1% changed the face mask after 2–3 h of wearing, whereas 36.9% wore a disposable face mask for more than 3 h in 1 day. A quarter of the respondents (24.9%) wore a disposable face mask for more than 1 day, with 44.4% of them wearing it for more than 3 h daily. Some of the respondents (13.0%) decontaminated their disposable face mask and reused it (χ^2^ = 255.499, *p* < 0.001).

Most cloth face mask users (93.7%) decontaminated their face masks before reusing, but only 55.7% of respondents did so on a daily basis. The respondents used the following types of decontamination for reusable face masks: washing (57.3%), washing followed by ironing (34.7%), disinfecting (8.0%) (χ^2^ = 41.16, *p* < 0.001).

**Figure 1:**
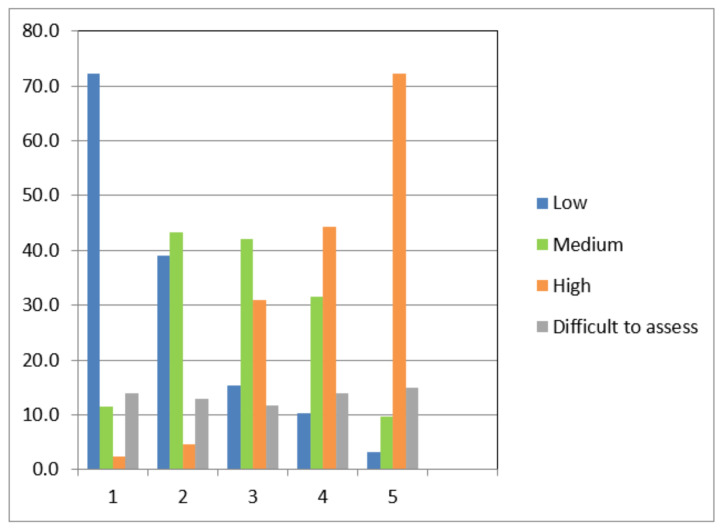
Subjective assessment of the effectiveness of different respiratory protective equipment (percentage distribution of responses). 1—single-layer face masks, 2—two-layer face masks, 3—three-layer face masks, 4—multilayer fabric face masks, 5—FF2/FF3 respirators.

The respondents’ subjective assessment of the effectiveness of different types of RPE was carried out on the following scale: low, medium, high. The results showed that the majority of respondents (72.3%) considered the single-layer face mask to have a low effectiveness (Figure 1). Respirators were considered the most effective by 72.2% of those surveyed. It should be noted that about 15% of the respondents found it difficult to assess the effectiveness of RPE.

We analyzed the observance of the face mask regime both by the respondents themselves in various places of mass gathering (Table 2) and their subjective assessment of the observance of the face mask regime by others (Table 3).

According to the results of the survey, the places where people stayed were ranked. Respondents most often wore face masks in medical institutions (91.5%), whereas the location with the lowest proportion of respondents wearing face masks was private transport (9.7%).

About one and a half percent of the respondents (1.4%) did not use RPE in any of the listed places.

When analyzing the subjective assessment of respondents of the observance of the face mask regime by others, the following was revealed. Most of the respondents believed that the face mask regime is more strictly observed in medical organizations (more than 70% of visitors wear face masks); the subway came in second (46.2% of respondents indicated that more than 70% of passengers observe the face mask regime). In other places, according to the respondents, visitors observe the face mask regime less often: more than 50% of visitors wear face masks in grocery stores and pharmacies, surface urban and suburban transport, and nonfood trade facilities.

## 4. Discussion

No statistically significant gender differences in the respondents’ answers were found, which is consistent with our earlier pilot study [34] and with literature data [35]. At the same time, many authors note that men are less likely to wear face masks [36] due to their typically risky behavior in relation to their health [25], associated, among other things, with the traditional role of men in society [26].

Almost all of the surveyed medical students wore RPE during the pandemic (97.5%), which is comparable to the data of other authors. The majority of other inhabitants surveyed in Bangladesh (98.7%), 71.2% of whom were students, wore face masks [37]. This was 99.5% in Vietnam [38] and 95.0% in the United Arab Emirates [39]. At the same time, only 62.8% of the medical students surveyed in Poland wore face masks [36], 71% in Pakistan [40], 69% in Congo [9], and 58.1% in Nigeria [41]. Some researchers did not evaluate the respondents’ wearing of masks, but rather their knowledge that a mask should be worn. This indicates the willingness of respondents to use face masks to reduce the risk of spreading coronavirus infection. Thus, according to Esmaeelinejad M. et al. [42], only 60.5% of Iranian dental students believe that wearing face masks is an effective measure for the prevention of COVID-19, while 100% of medical college students in China consider face masks to be effective [43].

The motivation for wearing the RTE for the majority of the respondents (75.8%) was a mask regime in the region of residence. That is, in the context of the pandemic, wearing face masks has become a social norm. Slightly more than half of the respondents put on face masks, realizing the risks of a pandemic (55.5% when it is impossible to maintain social distance with other people, 51.3% when the general epidemic situation in the city/country worsens, 15.0% when they come into contact with people who have obvious signs of the disease). The same trends were observed in a study by Irfan M et al. [14].

The majority of the respondents (73.7%) used one piece of RPE and, most often, disposable medical and hygienic two- and three-layer face masks. Taking into account the analysis of the selection criteria for RPE, these products were probably more comfortable for students. The same trend was noted by other researchers [36,44,45]. Nevertheless, a quarter of the students incorrectly affixed their face masks while wearing them—i.e., they did not cover the nose or moved them to the neck. Polish researchers also note that a third of the students surveyed affixed face masks incorrectly while wearing them [36]. Many of them (74.9%) reported exceeding the recommended time for wearing disposable face masks (2–3 h), and 13.0% even reused disposable face masks. About half (44.3%) of users of reusable face masks did not decontaminate them daily. Only 34.7% of respondents washed and ironed their face masks every day, following all the rules. According to MacIntyre C.R. et al. [46], only 6.8% of students wash their face masks on a daily basis. A properly treated cloth face mask can be just as effective as a medical mask, but according to WHO recommendations, it is recommended to wash cloth face masks using soap or detergent in hot water (at least 60 °C) at least once a day [9]. The low percentage of students properly following the WHO recommendations may be due to an underestimation of the personal danger, as well as incompetence in these matters. The lack of awareness of the respondents is probably due to the lack of interest of the majority of respondents (74.9%) in the information provided by the manufacturer of the RPE, which outlines, among other things, the wearing time and decontamination rules.

We have identified a different frequency of wearing face masks by students in different public places: face masks were found to be worn most often in medical organizations, grocery stores, and pharmacies, as well on the subway; the opposite was found to be true for suburban electric transport, taxis, and elevators. This may be due to both stricter control methods and students’ perceptions of different levels of risk of contracting COVID-19 infection in different places. In addition, we asked students to evaluate how they think the mask regime is observed by people around them in public places. In general, respondents felt that the people around them were less likely to wear face masks than they were. This is consistent with data from American scientists, who also found that young people, on average, underestimate the extent to which other people adhere to recommended preventive measures [47].

Most students (85.2%) reported using face masks at university. The reasons for not using face masks can be, as shown in our pilot study, as well as according to Matusiak, Ł. et al. [48], as follows: difficulty breathing and seeing due to the fogging of glasses, restriction of personal freedom, sweating, slurred speech, and irritation and itching of skin on the face [49]. All of these reasons make it difficult to use face masks while studying.

Knowing and correctly using RPE is important not only in terms of reducing the risk of infection, but also reducing the level of health concern and anxiety. We did not question participants regarding their psychophysiological state in this study, but researchers from China found that knowledge regarding means of protection, along with prognosis of the epidemiological situation and preventive measures, are protective factors against health concerns [50].

## 5. Conclusions

This study made it possible to identify the strengths and weaknesses of the practice of using face masks by medical students during the pandemic. Most of the respondents used RPE during the pandemic. However, not everyone did so correctly: the recommended wearing time was not observed, repeated use of disposable masks was practiced, and the mask was incorrectly affixed on the face. In overall daily life, students mostly wear medical masks. Despite the fact that masks are available to all students in Russia (in terms of price, assortment, and availability in stores/pharmacies), and employers and university management provide students with personal protective equipment during clinical practice, the effectiveness of this infection control measure is reduced if masks are used incorrectly. In order for medical students to be fully active participants in the fight against the pandemic, their knowledge and skills must be enhanced. In this regard, we consider it necessary to introduce a special module on nonspecific prevention into the training program for doctors. This module should include sections on the selection, use, handling, and disposal of protective equipment; changing behavioral patterns in accordance with the current epidemiological situation; and the importance and necessity of collective protective measures for the population during epidemics/pandemics. The information obtained should contribute to the development of expertise, i.e., the ability and readiness to implement a set of measures aimed at preserving and strengthening health and including the formation of a healthy lifestyle, preventing the occurrence and/or spread of infectious disease. These recommendations are fully in line with the actions of the WHO, whose academy offers a special training course for medical workers on the use of personal protective equipment using augmented reality technologies [51].

Potential limitations and future research directions: We conducted the survey only among 3rd-year students. In future, it would be interesting to compare the knowledge and attitudes to wearing face masks of students in different years of study, since a dependence of use of face masks on age was revealed [40]. Potential limitations of this study may be the “professional readiness” and “education” of medical university students in comparison with other professional groups of students and their peers who do not receive professional education. Therefore, it would be interesting to compare the attitude and practice of wearing face masks among students of different, including nonmedical, specialties. We focused on wearing face masks only. The use and relations of other nonspecific protective measures—e.g., physical distancing, coughing and sneezing etiquette, regular hand washing and use of alcohol-containing hand sanitizer, disinfection of mobile devices, and preventive behavior in society (for example, avoiding going to public places, traveling on public transport, or going out of the area of residence during isolation)—can also be analyzed.

## Figures and Tables

**Table 1:** Characteristics of study participants.

Characteristic	Men (*n* = 244)	Women (*n* = 744)	All (*n* = 988)
**Age** range M ± SD	19–24 21.07 ± 0.64	19–24 21.06 ± 0.59	19–24 21.06 ± 0.61
**Occupation (%)** 3rd-year student at medical university	100 (*n* = 244)	100 (*n* = 744)	100 (*n* = 988)
**Place of residence (%)** Moscow Moscow region other regions of the Russia	71.32 (*n* = 174) 14.34 (*n* = 35) 14.34 (*n* = 35)	69.75 (*n* = 519) 18.15 (*n* = 135) 12.1 (*n* = 90)	70.14 (*n* = 693) 17.21 (*n* = 170) 12.65 (*n* = 125)

M, mean; SD, standard deviation.

**Table 2:** Observance of the face mask regime by the respondents in different places where people stay.

Public Places	Number of Respondents Who Always Wear Face Masks in Different Places, in % (CI)
Medical organizations	91.5 (89.4–93.2)
Grocery stores/pharmacies	88.3 (85.9–90.3)
Subway	86.3 (84.0–88.4)
University	85.2 (82.8–87.3)
Surface urban transport	67.4 (64.4–70.3)
Nonfood trade facilities	59.1 (55.9–62.4)
Suburban electric transport	54.2 (50.1–57.3)
Taxi	46.6 (43.3–49.9)
Elevators	47.2 (44.0–50.3)
Outdoor entertainment, music, or sports activities	39.5 (36.5–42.7)
Indoor communication with friends or relatives	19.0 (16.7–21.7)
City park	14.0 (11.9–16.3)
Outdoor communication with friends or relatives	12.8 (10.9–15.1)
Private transport	9.7 (7.9–11.9)

CI, 95% confidence interval.

**Table 3:** Subjective assessment by the respondents of the observance of the face mask regime by visitors of various places of mass gathering.

Public Places	The Number of Respondents Who Noted the Observance of the Face Mask Regime by More Than 70% of People Around, in % (CI)
Medical organizations	68.2 (65.8–71.6)
Subway	46.2 (42.3–50.2)
Grocery stores/pharmacies	37.9 (34.6–40.8)
Surface urban transport	24.2 (20.9–27.8)
Suburban electric transport	17.4 (14.6–20.7)
Nonfood trade facilities	18.8 (16.5–21.4)

CI, 95% confidence interval.

## Data Availability

The data presented in this study are openly available in FigShare at https://doi.org/10.6084/m9.figshare.14686743 (accessed on 16 April 2021).
